# Primary Large-Cell Neuroendocrine Carcinoma of the Breast

**DOI:** 10.3390/diagnostics14212347

**Published:** 2024-10-22

**Authors:** Bicheng Zhang, Fengbo Huang, Siyu Guo, Dang Wu, Xiaofang Xiao, Ting Zhang

**Affiliations:** 1 Department of Radiation Oncology, The Second Affiliated Hospital of Zhejiang University, School of Medicine, Hangzhou 310009, China; bicheng_zhang@zju.edu.cn (B.Z.);; 2 Cancer Institute (Key Laboratory of Cancer Prevention and Intervention, National Ministry of Education), Second Affiliated Hospital, School of Medicine, Zhejiang University, Hangzhou 310009, China; 3 Department of Pathology, The Second Affiliated Hospital of Zhejiang University, School of Medicine, Hangzhou 310009, China; 4 Department of Medical Oncology, The Fourth Affiliated Hospital of Zhejiang University, School of Medicine, Yiwu 322000, China; xxfang2017@zju.edu.cn

**Keywords:** breast neuroendocrine carcinoma, large-cell neuroendocrine carcinomas, CgA, Syn, INSM1

## Abstract

Breast neuroendocrine carcinoma (NECB) is a rare type of breast tumor. Large-cell neuroendocrine carcinomas of the breast (LCNECB) are a special and rare histological subtype of NECB. Here, we present a case of a 59-year-old woman who was diagnosed with an LCNECB. A mass in the upper outer quadrant of the right breast was revealed via imaging. A histological examination showed the tumor cells were composed of clusters of large cells with obvious atypia that were polygonal or irregularly shaped. The patient underwent a right-breast-conserving radical surgery and sentinel lymph node biopsy (SLB). A histopathological examination revealed that the tumor of the right breast was 2.5 × 2 cm in size with vascular invasion, and the sentinel lymph node was negative. The immunohistochemical results showed that the tumor cells were diffuse and positive for chromogranin A (CgA), synaptophysin (Syn), and INSM1. The patient successfully completed chemotherapy and radiotherapy and is currently undergoing endocrine therapy.

Breast neuroendocrine carcinoma (NECB) is a rare type of breast tumor that accounts for less than 5% of breast cancers [1,2,3,4]. NECB was first endorsed as a distinct form of breast cancer by the World Health Organization (WHO) in 2003. NECB was divided into three histological subtypes, including well-differentiated neuroendocrine tumors, poorly differentiated or small-cell NECB, and invasive breast carcinoma with neuroendocrine differentiation. However, in the 2019 World Health Organization classification of tumors of the breast, primary breast neuroendocrine neoplasms are classified as a new type of pathological classification of breast cancer and are different from invasive breast cancer with neuroendocrine differentiation. But it must be emphasized that true primary neuroendocrine tumors of the breast remain uncommon and poorly defined [5]. Large-cell neuroendocrine carcinomas of the breast (LCNECB) have been much less frequently reported and have been described as other special and rare histological subtypes [6,7]. A pathological diagnosis of NECB should be confirmed immunochemically and should show positive results for neuroendocrine markers [8]. Neuron-specific enolase (NSE), chromogranin A (CgA), and synaptophysin (Syn) are the most sensitive and specific markers. INSM1 is a novel biomarker that shows potential as an accurate indicator of NECB diagnoses [9,10,11]. Estrogen receptors (ERs) and progesterone receptors (PRs) were reported to be expressed in 90% and 83% of NECB, respectively. [12,13] However, C-erbB-2 expression is rarely expressed in NECB [10]. LCNECB is similar to neuroendocrine markers in terms of immunochemical positivity, as described above, but it shows more specific cytologic features: a large cell size, a polygonal shape, a low nuclear–cytoplasmic ratio, a finely granular eosinophilic cytoplasm, occasionally prominent nucleoli, peripheral palisading, mitosis, and necrosis [14]. Previous studies on the prognosis of NECB have yielded contrary results due to different diagnostic criteria and the limited number of cases. As a very aggressive tumor, LCNECB shows highly invasive behavior, has a strong tendency towards distant metastasis and local recurrence, and has a poor prognosis. Fortunately, in this article, the patient’s breast cancer was found at an early stage. There is no standard treatment of NECB due to the limited number of cases and the lack of clinical trials. The most common current treatments of NECB are mainly for ductal carcinoma [7,15]. Molecular analysis is critical. It is worth mentioning that the ER and PR are often highly expressed in NECB. Endocrine therapy has a definitive effect in treating luminal-like breast cancer. However, due to the low expression of C-erbB-2 in NECB, anti her-2 therapy is difficult to apply in the vast majority of patients [15].

**Figure 1:**
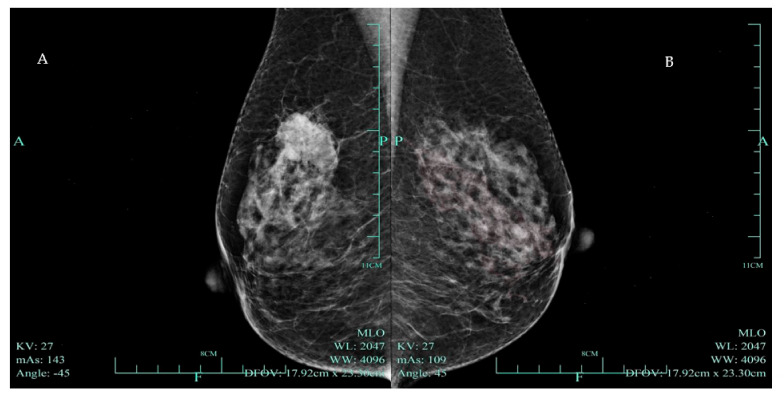
(**A**) Mammogram of a hyperdense mass highly suggestive of malignancy (BI RADS—4C) in the right breast. A 59-year-old woman had had a mass in the upper outer quadrant of her right breast for 2 weeks. Physical examination revealed an unfixed hard mass in the right breast. Mammography demonstrated an irregularly shaped high-density mass with boundary burrs in the upper outer quadrant of the right breast, with a maximum diameter of 33 mm, highly suggestive of malignancy (BI RADS—4C). (**B**) A normal mammogram of the patient’s left breast.

**Figure 2:**
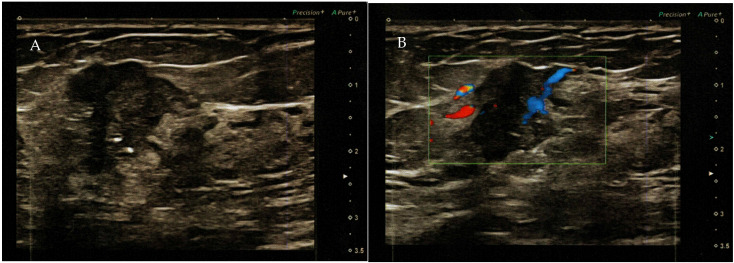
(**A**) Color ultrasound revealed a nodule in the right upper outer quadrant of the breast with disordered structure, increased echogenicity, uneven distribution of mammary glands, unclear boundary, irregular shape, and a strong echogenic spotted hypoechoic nodule with a diameter of 2.2 × 1.4 cm (BI RADS—4B). (**B**) Mixed blood flow signals in the tumor.

**Figure 3:**
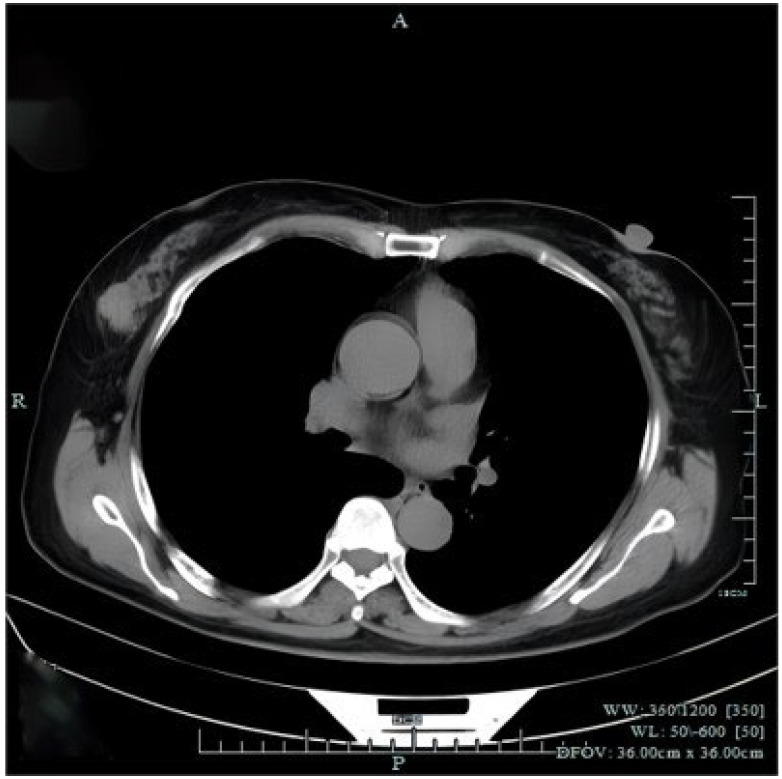
Chest computed tomography (CT) revealed a mass in the right breast without axial or mediastinal lymph node swelling. There were no abnormal findings observed in distant organs from abdominal CT and cranial magnetic resonance imaging. The patient underwent right-breast-conserving radical surgery and sentinel lymph node biopsy (SLB). Histopathological examination revealed that the tumor of right breast was 2.5 × 2 cm in size with vascular invasion and the sentinel lymph node biopsy was negative.

**Figure 4:**
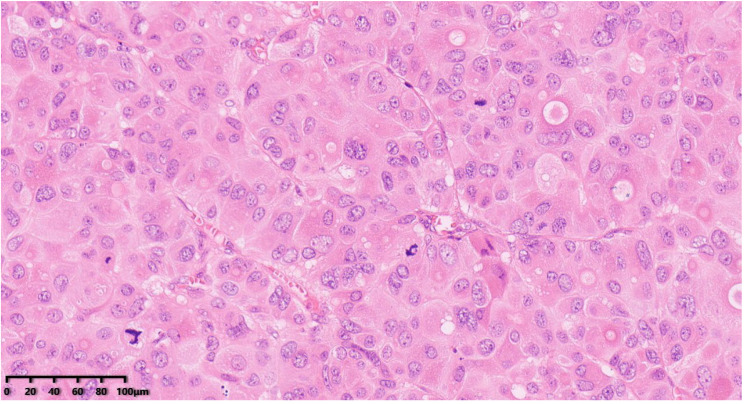
Histological examination showed the tumor cells were composed of clusters of large cells with obvious atypia that were polygonal or irregularly shaped. The cytoplasm of the tumor cells was abundant, eosinophilic with obvious nuclear atypia, obvious nucleolus, active mitotic image (Figure 4, 200×). Hematoxylin–eosin staining was used.

**Figure 5:**
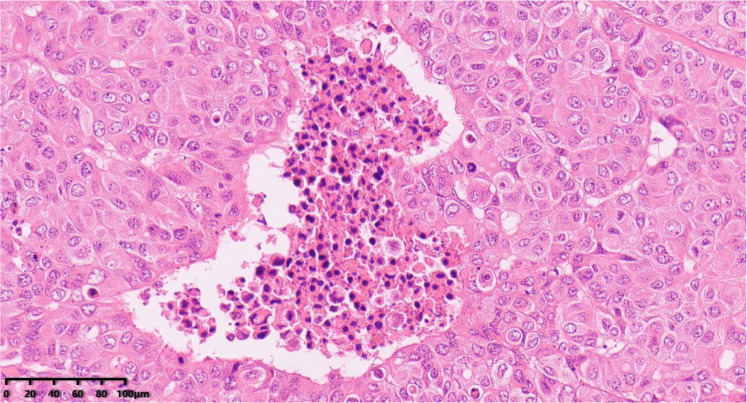
Focal necrosis (200×). Hematoxylin–eosin staining was used.

**Figure 6:**
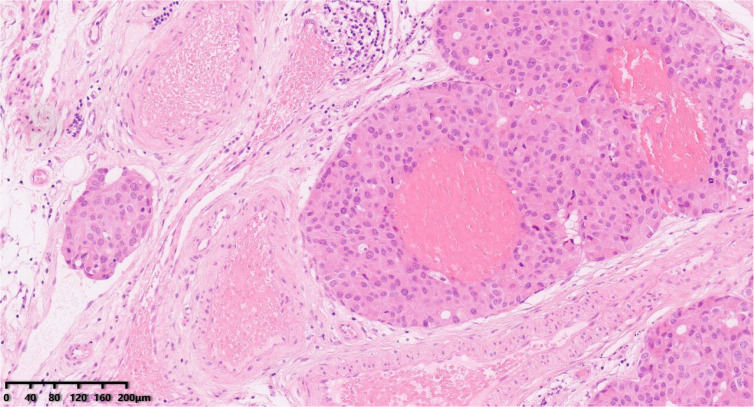
Vascular invasion (Figure 6, 100×). Hematoxylin–eosin staining was used.

**Figure 7:**
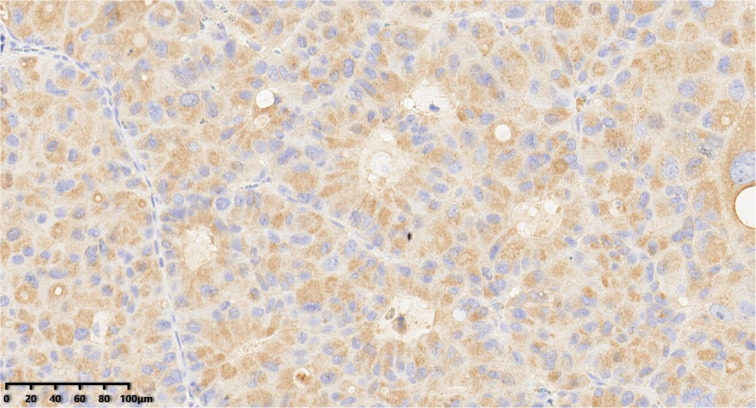
Immunohistochemical results showed that tumor cells were diffuse and positive for chromogranin A (200×). EnVision method was used.

**Figure 8:**
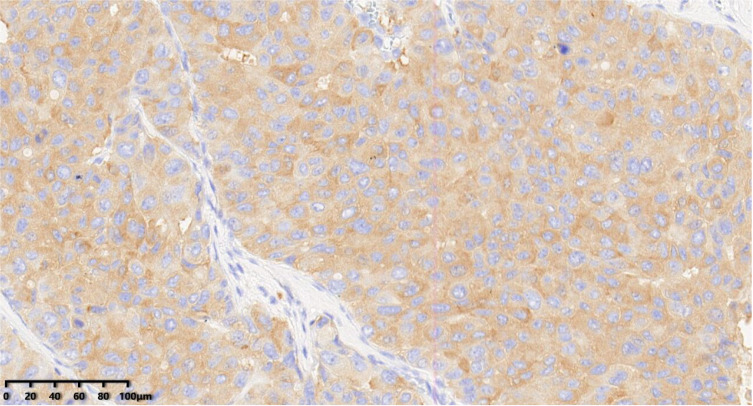
Immunohistochemical results showed that tumor cells were diffuse and positive for synaptophysin (200×). EnVision method was used.

**Figure 9:**
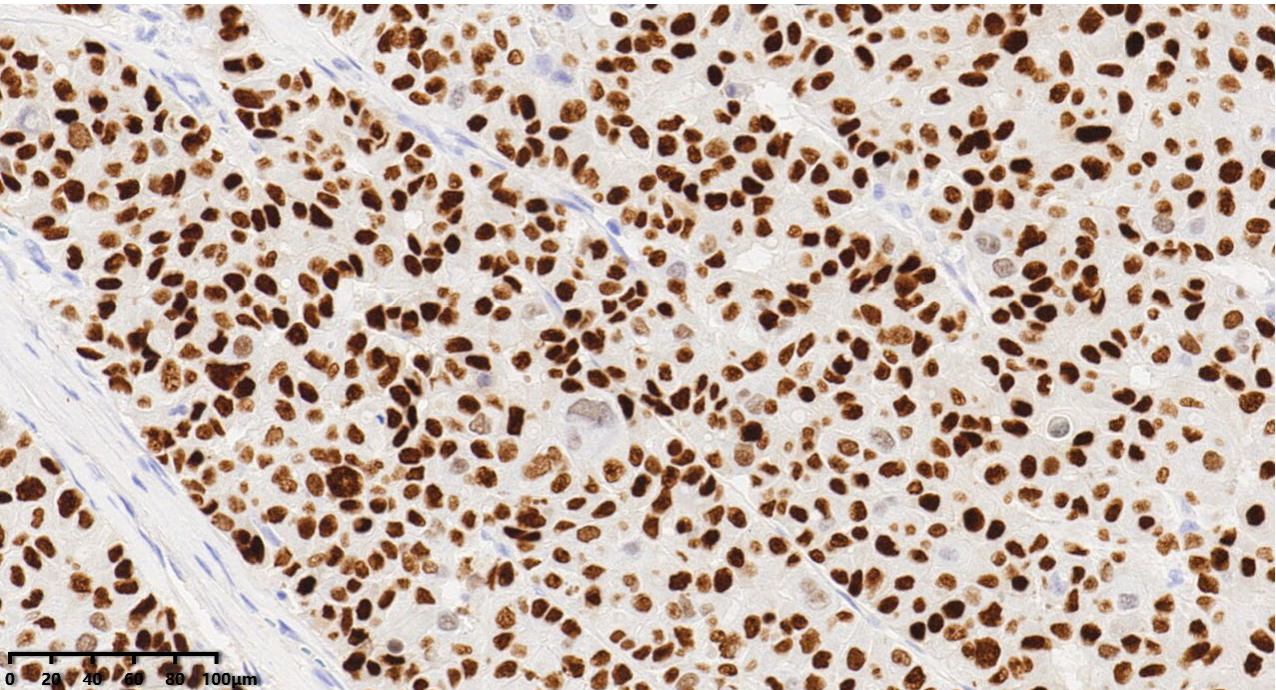
Immunohistochemical results showed that tumor cells were diffuse and positive for INSM1 (200×). EnVision method was used.

**Figure 10:**
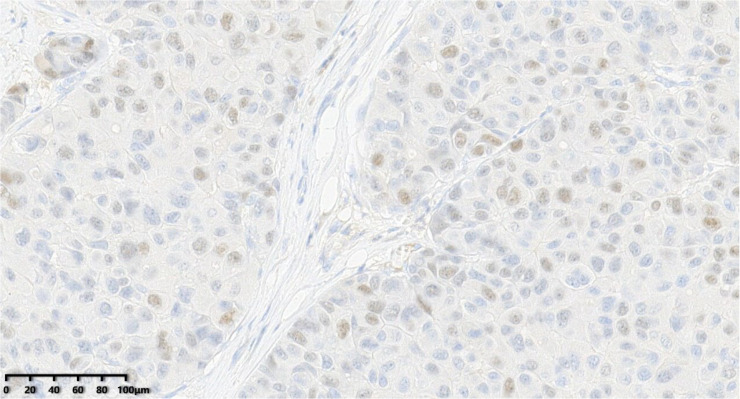
Thirty percent of the tumor cells were positive for ER (200×).

**Figure 11:**
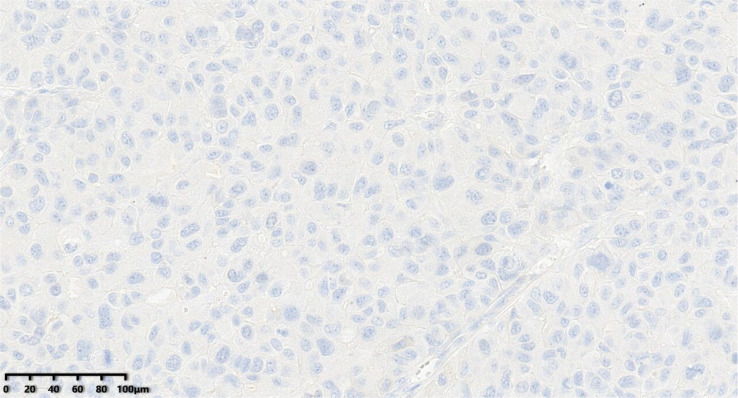
The tumor cells were negative for PR (200×).

**Figure 12:**
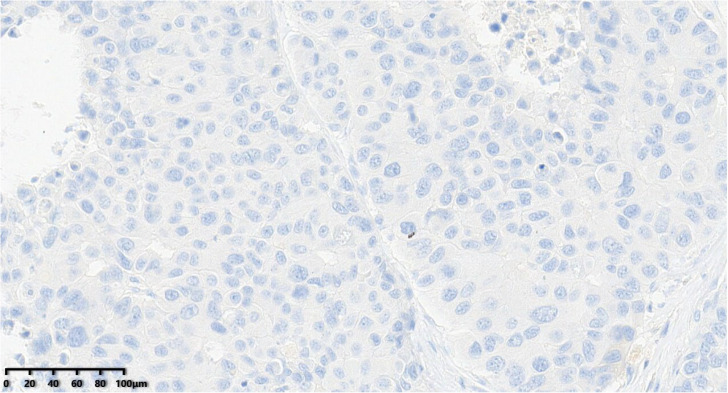
C-erbB-2 score of the tumor was 0 (200×).

**Figure 13:**
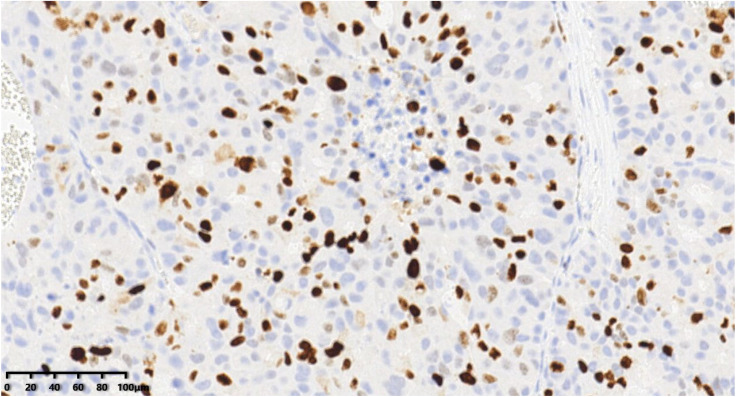
ki-67 proliferation index of the tumor was about 40% (200×).

**Figure 14:**
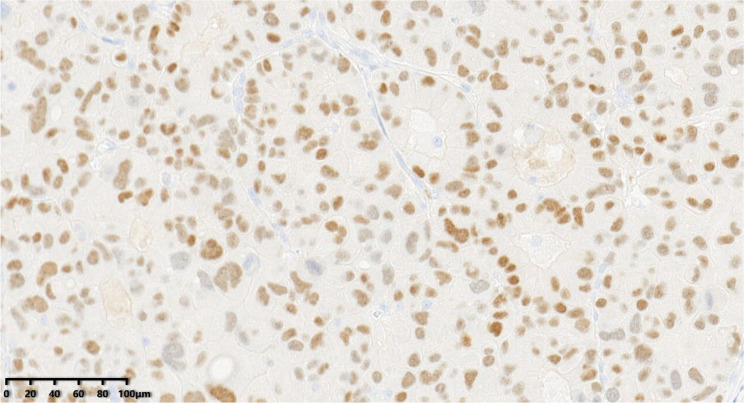
The tumor cells were positive for GATA3 (200×). According to the immunohistochemical examination (IHC), the patient was diagnosed with grade III large-cell neuroendocrine carcinoma of the breast (LCNECB). EnVision method was used. After diagnosis, the patient received AC * four cycles of adjuvant chemotherapy, radiotherapy, and endocrine therapy. The patient successfully completed chemotherapy and radiotherapy, and is currently undergoing endocrine therapy.

## Data Availability

Data are contained within the article.
